# Factors affecting the choice of medical specialties in Turkiye: an analysis based on cross-sectional survey of medical graduates

**DOI:** 10.1186/s12909-024-05349-7

**Published:** 2024-04-04

**Authors:** Mustafa Said Yıldız, M. Mahmud Khan

**Affiliations:** 1 Ministry of Health– Türkiye, Ankara, 06630 Turkey; 2 Department of Health Policy and Management, College of Public Health, University of Georgia, 116 Wright Hall, Health Sciences Campus, 100 Foster Road, Athens, USA

**Keywords:** Factors affecting choice of medical specialization, Malpractice risk, Workplace violence, Medical specialties

## Abstract

**Background:**

Modern healthcare systems require the right mix of medical specialties for effective provision of high-quality services. Despite increased availability of general physicians and specialists, Türkiye lags behind high-income countries in terms of availability of specialists. The purpose of the study is to identify several specific factors that affect the choice of medical specialization.

**Methods:**

All 350 medical school graduates in a specialty examination preparation bootcamp were requested to participate in the survey and 333 completed the self-administered questionnaire. The survey asked questions about factors affecting choice of medical specialty by medical graduates.

**Results:**

The empirical results indicate that surgical specialties, compared to other broad medical specializations, are selected because of its higher income-earning potential and social prestige. The likelihood of selecting surgical specialties is negatively affected by rigorousness of the training program, high work-load, risk of malpractice lawsuits and risk of workplace violence. Male participants were 2.8 times more likely to select surgery specialty compared to basic medical science. Basic medical science areas were selected at a higher rate by female graduates and graduates with high level of academic performance in medical schools.

**Conclusions:**

It is critically important to improve trust and inter-personal communications between the patients and physicians in all specialties to lower the likelihood of malpractice lawsuits and workplace violence. Policy-makers may adopt policies to affect income earning potential and social prestige of targeted specializations to improve their supply.

## Introduction

Modern healthcare systems require the right mix of clinical specialties to improve quality of healthcare services and the health status of population. With epidemiologic transition, economic progress and technological development, the degree of specialization among healthcare providers increase and consumers tend to increase the demand for highly specialized services. Medical specialists often plan, supervise and evaluate the implementation of care, conduct trainings, carryout research to further improve technical quality and effectiveness of healthcare services. Therefore, investment in medical specialization improves healthcare delivery system, enhance technical innovation and improve quality and effectiveness of specialized medical services and interventions [1].

Despite the increasing demand for medical specialists, several specialist-types are in short supply in some countries of the world, implying that it is important to identify the barriers to entry to medical specializations, especially for the ones that are likely to see rapid increase in demand with improved economic status and ageing of the population. Health systems need to develop and implement policy options for encouraging physicians to select the medical specializations society will need in the near future. In Turkiye, medical specialization includes specialists as shown in Table 1. Note that general practice is not considered a medical specialization.

In the last few decades, Türkiye has seen significant improvements in healthcare delivery with improved service quality and access to care. The Health Transformation Program of 2003 accelerated improvements in population health, geographic distribution of health services and healthcare coverage by reforming social security and strengthening the health infrastructure and management. Lack of specialists was considered as one of the main concerns during the implementation of Health Transformation Program. To address this concern, Türkiye adopted the strategy of increasing the training slots in the existing specialty residency programs and establishing new residency programs in medical schools [2]. The number of specialists per 100,000 population increased in the country from 68 in 2002 to 105 in 2020 but still, Türkiye lags behind high-income countries of the Organization for Economic Cooperation and Development [3].

The availability of residencies by medical specializations and preference patterns of graduating medical students determine the distribution of specialist-types trained. A good system of matching medical graduates with their preferred medical specialties should improve job satisfaction and career development. Ensuring training of adequate number of medical specialists consistent with the needs of the population should improve access to specialized care and overall effectiveness of health care system. In addition to general shortages of medical specialists, a lack of alignment between health system needs for specialized medical care and specialty preference of medical graduates has been a problem for several countries of the world [4]. Avoidance of specific specialties by medical graduates due to factors other than societal needs may further amplify physician maldistribution by geographic location and mix of specialties [5]. Even in countries with relatively high availability of specialists, some specific specializations are in short supply [6]. France, for example, shows shortages of obstetrics and gynecology while pediatric surgery, anesthesiologist, orthopedics are in short supply in Spain [7].

It has been well documented that earning potential as well as social recognition and reputation encourage the choice of medical specialty but there are many other factors that influence the decision-making. In recent years, healthcare providers are facing increasing risk of workplace violence [8]. Violence against healthcare workers has become an important problem in the Turkish healthcare system as well. Consistent with the definition adopted by the National Institute for Occupational Safety and Health (NIOSH), the survey defined violence as “physical and verbal violence”. This does not include sexual violence. Violence against healthcare providers imply “client-on-worker violence” not worker-on-worker violence, family dispute-related violence or interpersonal violence due to criminal activity unrelated to the profession. Since not all medical specialties are equally susceptible to workplace violence, increasing risk of violence may influence choice of certain high-risk medical specialties. Anecdotal evidences suggest that the likelihood of facing violent encounters with patients and/or their family members have affected selection of medical specialties in Türkiye [9]. Another related factor is the intensity and severity of medical malpractice lawsuits faced by different medical specialists. Risk-averse individuals may prefer to select relatively low-risk specialties [10]. Studies evaluating choice of specialties in Türkiye suggest that the perception of increasing violence against healthcare professionals and medical lawsuits discourage medical graduates from selecting surgery-related specialties [11].

Selection of medical specialties is also affected by medical graduates’ self-appraisal of their abilities, skills and aptitudes [12, 13], financial expectations [14], prestige of the specialty [15], career development opportunities [16], work load during residency [17] length of residency years [18, 19] and opportunity to teach [20]. A validated instrument included patient care characteristics (multidisciplinary, acute and continuous) and specialty characteristics (challenging nature, surgical based) as additional factors in the choice of medical specialization [21]. A scoping study on the choice of specialty education by medical students in low- and middle-income countries listed “immigration opportunities” as an important factor [22]. Medical graduate’s preference for primary care over advanced care also affect type of specialties chosen later on [23]. Only a limited number of analyses included factors that negatively affect the choice of specialization such as malpractice risk, risk of legal complaints and low level of satisfaction in professional life [18, 24, 25]. Violence towards physicians has also been examined as a potential factor in recent years [26, 27]. This study intends to examine various positive and negative factors, including violence against healthcare providers and malpractice risk, in the choice of medical specialties.

## Materials and methods

### Study design

This study was a cross-sectional study of medical graduates.

### Study setting

A survey was carried out among the participants of a bootcamp that helps medical graduates to prepare for their residency entrance examinations. The bootcamp selected for the study was located in Ankara. Bootcamps are usually offered in larger cities and trainees join the bootcamps from all over the country. 350 medical school graduates were getting training in this bootcamp at the time of the survey.

### Survey procedure

A survey instrument was designed and distributed to all 350 medical graduates in the bootcamp and 333 completed the survey. Participation in the survey was completely voluntary. The sample size was adequate for estimation of mean values of different variables of interest. At 95% confidence level with precision of 5%, the required sample size turns out to be 250. The structured instrument was self-administrated by the participants. The survey in the bootcamp was carried out in February 2022.

### Study questionnaire

The survey questionnaire asked the respondents to report their gender (male or female), whether they were married or not, years since graduation from medical school, academic performance in medical school, years in practice if they practiced medicine prior to taking the specialty examinations. Specialty entrance examination selected by a respondent was considered the medical specialty chosen. The respondents were also asked to indicate their subjective evaluation of the degree of importance of various factors affecting choice of medical specialization. The list of factors influencing the choice of specialty were obtained from studies carried out by Harris et al. and Chang et al. [12, 15] and few additional factors, such as, malpractice risks and violence towards healthcare professionals were added in the survey. Participants were asked to rate the importance of the factors using a 5-point Likert scale, 1 being not important and 5 being highly important.

Since physicians can select residencies from a long list of possible specializations, we have grouped these into three broad types: basic medical science, internal medicine and surgery. Table 1 lists the specific medical specializations that were included in each of the three categories.

**Table 1:** Specific clinical specializations used to define the broad specialization-types in the survey of medical graduates

Surgery specialty-type	Internal Medicine Specialty-type	Basic Science Specialty-type
Emergency medicine General surgery Orthopaedics and traumatology Plastic, Reconstructive and Aesthetic Surgery Eye diseases Anaesthesiology Neurosurgery Paediatric surgery Gynaecology and obstetrics Cardiovascular surgery Otolaryngology Urology	Chest Diseases Radiology Mental Health Internal Medicine Child Health and Diseases Paediatrics Paediatric Mental Health Skin and Venereal Diseases Cardiology Infectious Disease and Clinical Microbiology Physical Medicine and Rehabilitation, Neurology	Anatomy Public Health Histology and Embryology Medical Biochemistry Medical Pharmacology Medical Genetics Medical Microbiology Medical Pathology Physiology

### Data analysis

To describe the demographic characteristics and level of importance of various factors, mean values, differences in means and p-values of differences in means were calculated by using a set of regression equations with choice of specialty as the right-hand-side variables. The estimated equations can be written as follows:

$$ {y}_{ki}={\alpha }_{k1}+\sum _{j=2}^{T}{\beta }_{kj}{S}_{ij}+{u}_{ki}$$, where $$ {y}_{ki}$$ is the $$ {k}^{th}$$ variable for individual $$ i$$ and $$ k=1,\dots.v$$, $$ T$$ is the total number of specialty-types considered (three specialties in the analysis), and $$ \beta $$ are the estimated coefficients for specialization type $$ {S}_{j}$$ selected by respondent $$ i$$ and $$ {\alpha }_{k1}$$ is the mean value for the base specialization “1”. The estimated coefficients represent the differences in the mean values of $$ {k}^{th} $$and base specialty types. The p-values associated with the t-statistics of the coefficients indicate the statistical significance of the differences in the means. Note that, adding $$ {\alpha }_{k1}$$with $$ {\beta }_{kj}$$ will be the mean value of variable $$ k$$ for the specialty-type j.

The choice of specialty is a complex process of decision-making where many factors interact with each other to determine the specialty selected. To understand the effect of various covariates and factors affecting the choice, a multivariate regression model was employed. Since the purpose of the study is to understand the selection of one of the medical specialties out of three possible options, a multinomial logistic regression model was run to estimate the effect of various factors on the likelihood of selecting a specialty-type. For the multivariate regression analysis, the Likert Scale responses were categorized into low and high-level responses. If the Likert scale selected by a respondent was 4 or 5, it was recoded as 1 (i.e., the factor was highly important in the choice) and zero otherwise. Therefore, after the recoding of the Likert Scales, the variables indicate whether the specific factor was considered important in the choice of medical specialty by the surveyed individuals.

## Results

Table 2 reports the differences in mean Likert Scale values for the specialties using “basic science” specialty as the comparator. The t-statistics indicate statistical significance of the differences in the mean (t-value of 1.96 or higher implies significance at 5% level). The difference in mean scores were not significant between internal medicine and basic sciences for factors like earning potential, prestige of the specialty, future career prospect, opportunity for research, length of training, training program relatively easy and family expectations. The differences in mean values for surgery specialty and basic sciences were not statistically significant for future career prospect, opportunity for research, length of training and family expectations.

**Table 2:** Mean values for base specialty and differences in mean values for variables affecting the choice of specialties

Potential variables and factors affecting choice of specialty	Mean value for	Differences in the mean values of variables from basic medical science mean values	
	Basic medical science	Surgery and basic medical science (p value)	Internal medicine and basic medical science (p value)
Male (= 1)	0.417	0.157 (0.030)	-0.005 (0.939)
Married (= 1)	0.330	-0.066 (0.302)	-0.127 (0.025)
Graduated more than a year ago (= 1)	0.485	-0.198 (0.004)	-0.192 (0.002)
Personal preference for the specialty	3.650	0.752 (0.000)	0.573 (0.000)
Self-perceived personal competence in the specialty	3.602	0.685 (0.000)	0.496 (0.000)
Earning potential of the specialty	3.369	0.401 (0.011)	0.016 (0.910)
Prestige of a provider in the specialty	3.165	0.777 (0.000)	0.171 (0.253)
Risk of violence in the specialty	4.515	-1.641 (0.000)	-1.025 (0.000)
Future career prospect in the specialty	3.864	0.193 (0.190)	0.059 (0.652)
Opportunity for research in the specialty	3.534	0.144 (0.379)	0.081 (0.575)
Opportunity for serving the community	2.534	0.914 (0.000)	0.865 (0.000)
Malpractice and legal issues	4.388	-1.802 (0.000)	-0.780 (0.000)
Length of training in the specialty	2.350	-0.327 (0.069)	-0.035 (0.827)
Training program for the specialty relatively easy	3.262	-0.998 (0.000)	-0.339 (0.058)
Workload in the specialty	4.252	-1.586 (0.000)	-0.847 (0.000)
Family expectation	2.320	-0.185 (0.290)	0.134 (0.388)

* Information on each of the factors was collected on a five-point Likert scale and the mean values, in theory, will be in the range 1 to 5. The values reported in the table is the differences in the mean values of the factors for the specialty-type from the mean values for the base specialty-type. The t-values show the significance of the differences in means. The cut-off t-value for 5% precision-level is 1.96 in absolute value.

Although, the comparison of mean values provided some idea on the importance of each of the factors in the choice of medical specialty-type, it cannot indicate independent effect of each of the factors after controlling for other factors. In other words, in real life, decision-making is based by considering all the potential factors taken together. As mentioned in the method section, we defined three specialty-types to identify factors affecting the choices compared to the “base specialty”. In our model, “basic medical science” was considered the base specialty to estimate a multinomial logistics model. The results of the multinomial logistic regression are reported in Table 3.

The results suggest that male respondents were more likely to select surgery specialties compared to basic science. If the respondent graduated more than a year ago at the time of specialization examination, he/she was less likely to select either the internal medicine or surgery compared to the base specialization. The concern about risk of violence against the healthcare providers lowered the likelihood of selecting internal medicine and surgery. Another factor that negatively affected the choice of surgery was the risk of malpractice and legal issues. Income earning potential was not significant in the choice of internal medicine specialization compared to basic clinical sciences but it was significant in the choice of surgery. Family influence or expectations affected the likelihood of selecting surgical specialties but it had no effect on the choice of internal medicine compared to basic medical science specialization. Self-perceived competency in medical fields positively affected the choice of internal medicine and surgery specializations.

**Table 3:** Results of multinomial logistic regression for factors affecting the choice among three clinical specialties (with “basic science’ as the base outcome)

Clinical specialization selected and factors affecting choice of specialty	Relative risk ratio	Standard error	Z	P > z
Basic medical sciences (base outcome)				
***Internal medicine***				
Male	1.406	0.451	1.060	0.288
Graduated more than a year ago	0.496**	0.159	-2.180	0.029
Self-perceived competency is important	2.308**	0.808	2.390	0.017
Ability to serve the community by specialty selection is important	2.924**	0.966	3.250	0.001
Family expectation on selection of specialty is important	2.009*	0.828	1.690	0.091
Income earning potential is important	1.164	0.368	0.480	0.631
Potential risk of violence against healthcare providers important	0.127**	0.058	-4.550	0.000
High level of performance in medical school	0.835	0.267	-0.560	0.572
Risk of malpractice and other legal issues important	0.753	0.308	-0.690	0.487
Constant term (baseline Relative Risk Ratio)	2.892**	1.373	2.240	0.025
***Surgery***				
Male	2.886**	1.107	2.760	0.006
Graduated more than a year ago	0.377**	0.148	-2.480	0.013
Self-perceived competency is important	5.782**	2.879	3.520	0.000
Ability to serve the community by specialty selection is important	2.338**	0.919	2.160	0.031
Family expectation on selection of specialty is important	2.695**	1.297	2.060	0.039
Income earning potential is important	2.385**	0.920	2.250	0.024
Potential risk of violence against healthcare providers important	0.110**	0.056	-4.300	0.000
High level of performance in medical school	0.341**	0.148	-2.470	0.013
Risk of malpractice and other legal issues important	0.254**	0.115	-3.020	0.003
Constant term (baseline Relative Risk Ratio)	0.902	0.546	-0.170	0.865

Multinomial logistic regression model: Number of observations = 333, LR χ^2^(18) = 160.93, Prob > χ^2^ = 0.0000, Log likelihood = -278.047, Pseudo R^2^ = 0.2244

** Significant at 5% level or better

* Significant at 10% level

## Discussions

More than 40% of surveyed medical graduates in Türkiye preferred internal medicine as their specialization while surgery was selected by less than 30% of graduates. Surgery was more common among male medical graduates compared to other two broad specializations considered. In 2021 residency examinations, 19 female and 47 male graduates were placed to cardiovascular surgery residency, 47 female and 136 male to general surgery, 22 female and 66 male to neurosurgery indicating preference of male physicians in surgery-related specializations [22]. About 39% of male medical graduates selected internal medicine as their area of specialization while about a third selected surgery. When the mean values of potential factors affecting specialization were considered, all the mean values were found to be highly significant. We expected “personal preference” index to be close to 5.0 but the values were significantly lower for both the basic medical sciences and internal medicine. This probably implied that medical graduates did not select a specialization based on their own personal preferences.

The multinomial regression model indicated the relative importance of different factors in the choice of a specialty relative to their effects on reference specialty, basic medical science specialty-type. Male medical graduates were more likely to select surgery compared to its effect on the selection of other two specialty-types. Those who graduated from medical schools more than a year ago were less likely to select internal medicine and surgery. The delay in taking the specialization entrance examinations probably discouraged medical graduates to select these specializations which require higher overall scores in the examinations than the basic medical science. Self-perceived competence in medicine increased the likelihood of selecting internal medicine and surgery compared to selecting basic medical science. Income earning potential increased the likelihood of selecting surgery compared to basic sciences. Risk of violence against medical practitioners showed high negative effects on the choice of both internal medicine and surgery compared to basic sciences. Physicians involved with direct service provisions were more likely to face violence discouraging medical graduates to select internal medicine and surgery. Malpractice lawsuits and other legal concerns significantly lowered the likelihood of selecting surgery compared to basic sciences. Expectations of family members significantly influenced selection of surgery compared to basic sciences. Therefore, risk of facing violence and medical malpractice lawsuits showed high negative effects on the selection of surgery compared to basic sciences. Even the selection of internal medicine was negatively affected by these factors although not as strongly as in the case of surgery-related specialty type. Scott et al. found that compared with students interested in a career in family medicine, those interested in surgery were more likely to be influenced by prestige of the profession [28]. In this study, however, after controlling for other factors like income earning potential, prestige did not turn out to be important. Glynn and Kerin (2010) emphasized the importance of relatively easy lifestyle during training for not selecting surgery over other medical specializations [14]. In our multinomial logistic model, relatively easy training program (lower workload) showed no effect on the choice of medical specialties. Thornton (2000) found that economic incentives (and medical school indebtedness) and anticipated income affect choice of specialty [29]. Our results indicated that income earning potential of medical specialization increased the likelihood of selecting surgery compared to basic medical sciences and internal medicine.

Two factors influencing choice of specialty, opportunity for research and years required to complete training, had low Likert scores for general practice and other specialty trainees in Harris et al. (2005) study. These factors also did not show any statistically significant impact on the choice of medical specialties in our study. Contrary to the results reported in Harris et al. (2005), our study found that risk of malpractice significantly lowered the choice of surgery specialty compared to basic medical sciences and internal medicine, controlling for other relevant factors [15].

The multinomial logistic model indicated that the risk of medical errors, lawsuits and the risk of violence are highly significant in avoiding surgical specialty-type implying that the specialty-type may not remain highly sought-after specializations if these negative factors continue to increase over the years. There have been indications that surgery specialty-type is already showing reduced demand compared to basic sciences and internal medicine. Residency examination results appear to confirm this change in the selection of specializations [30]. Male medical graduates selecting surgery at a higher rate may also be related to risk of malpractice and violence because female professionals tend to be more sensitive to these negative situations. It is interesting to note that those who had better performance in medical school showed lower likelihood of selecting internal medicine or surgery as their specializations. It is possible that the medical students with significantly better academic performance were more interested in medical research and innovation and selected the basic science-related specializations.

Türkiye has seen declining interest in surgical specialties by medical graduates and this is clearly reflected by trend in specialty entrance exam scores and the demand for the specialization examinations. Lower proportion of medical graduates opted for examinations in surgery specialties and the cut-off scores for admitting medical graduates in surgery have been declining. Our results suggested that risk of malpractice and risk of violence are important in creating this lower interest in surgery.

The Ministry of Health in Türkiye has adopted policies to lower or prevent violence in healthcare settings. Establishing the violence reporting system (white code), offering legal assistance to victims of violence, adoption of severe punishment for perpetrating violence against medical professionals are some of the important policy options implemented. In May 2022, a law was enacted that allows the state to pay for malpractice compensation unless it is intentional. This law also included severe punishments for perpetrators of violence [31].

In order to improve safety and quality of healthcare services, which should reduce likelihood of violence and malpractice lawsuits in healthcare, structural reforms and system improvements need to be carried out. It is critically important to improve patient-physician trust and communications to lower the risk of malpractice and litigation. Efforts should also be made to improve clinical quality and patient outcomes in order to reduce violence against physicians. Improving clinical quality of care lowers the conflicts between the healthcare providers and patients and will help improve trust in physicians and the healthcare system.

The study has several important limitations. The survey was carried out in one bootcamp where medical graduates were taking preparations for their residency examinations. There were only 350 participants in the bootcamp although total number of students who take residency examinations in a year exceed 20,000. Therefore, the survey may not be fully representative of all test-takers in the country, although medical graduates from all over the country joins the bootcamps. In addition, not all test-takers attend bootcamps implying that the bootcamp participants may represent a biased sample because of self-selection bias. Those who participate in bootcamps may have high degree of motivation in passing the examinations and they may be economically better-off than the non-participants. This survey is a self-administered survey and bias in reporting is always a possibility. Another limitation is the grouping of diverse specialties into three specialty-types. This categorization fails to indicate how different factors affect the choice of highly specialized practice areas. Given the sample size, finer categorization of specialties was not feasible.

## Conclusion

Although medical school graduates choose surgical specialties more than other specialties because of their personal interest, competence and the specialties’ higher anticipated income, they show avoidance of these specialties because of risk of malpractice and risk of violence. When all the various factors were considered, these avoidance factors significantly lowered the likelihood of selecting surgery and internal medicine compared to basic medical sciences. The effect of these factors on specializations related to surgery is significantly higher than its effect on internal medicine. Policy-makers need to identify policy options for lowering the importance of the avoidance factors as well as reducing the degree of adverse effects created by these factors.

Although the results in this study are Türkiye specific, these may have wider applicability. Income-earning potential is an important driver of selecting medical specialization but, the analysis indicated that social prestige of the specialty, family expectations and self-perceived competency may also affect the choices. Therefore, potential income is not the sole or even the most important determinant of medical specialty selection and factors like social prestige, self-perceived competency can potentially be influenced in any country to guide medical school graduates’ choice of specialties. Violence against healthcare providers has been increasing around the world and this analysis implies that unless client-to-healthcare provider violence can be controlled, it will negatively affect choice of high-risk medical specialty, adversely affecting the health system and health outcomes.

## Data Availability

The dataset analyzed will be made available to all interested researchers from the first author of the paper.
